# Comparison of tocilizumab and tumour necrosis factor inhibitors in rheumatoid arthritis: a retrospective analysis of 1603 patients managed in routine clinical practice

**DOI:** 10.1007/s10067-015-2879-0

**Published:** 2015-01-29

**Authors:** Marina Backhaus, Jörg Kaufmann, Constanze Richter, Siegfried Wassenberg, Anne-Eve Roske, Peter Hellmann, Markus Gaubitz

**Affiliations:** 1 Medizinische Klinik mit Schwerpunkt Rheumatologie und klinische Immunologie, Charité-Universitätsmedizin Berlin, Charitéplatz 1, 10117 Berlin, Germany; 2 Praxis Dr. med Jörg Kaufmann, Ludwigsfelde, Germany; 3 Internistisch-rheumatologische Schwerpunktpraxis, Stuttgart, Germany; 4 Fachkrankenhaus Ratingen - Rheumatologische Klinik, Rheumazentrum Ratingen, Ratingen, Germany; 5 Roche Pharma AG, Grenzach-Wyhlen, Germany; 6 Chugai Pharma, Frankfurt, Germany; 7 Akademie für Manuelle Therapie an der WWU Münster, Interdisziplinäre Diagnostik und Therapie, Münster, Germany

**Keywords:** DMARDs, Patient-reported outcomes, Remission, Rheumatoid arthritis, Routine practice, Tocilizumab, Tumour necrosis factor inhibitors

## Abstract

Tocilizumab (TCZ) and tumour necrosis factor inhibitors (TNFi) are recommended for the treatment of rheumatoid arthritis (RA) in patients with inadequate response (IR) to prior disease-modifying antirheumatic drugs (DMARDs). This retrospective analysis assessed the efficacy of TCZ and TNFi, alone or in combination with DMARDs, in 1603 patients with IR to previous treatment with either DMARDs (DMARD-IR) and/or TNFi (TNFi-IR), initiating treatment with TCZ or a TNFi, managed in routine clinical practice. Patients were grouped according to treatment history and treatment initiated: DMARD-IR patients initiating treatment with TCZ + DMARD (DMARD-IR TCZ) or TNFi + DMARD (DMARD-IR TNFi), DMARD-IR and/or TNFi-IR patients initiating treatment with TCZ monotherapy (TCZ mono) or TNFi monotherapy (TNFi mono), and TNFi-IR patients initiating treatment with TCZ + DMARD (TNFi-IR TCZ) or TNFi + DMARD (TNFi-IR TNFi). Patients initiating treatment with TCZ generally had more severe disease and longer disease duration compared with the corresponding TNFi group. Significantly more patients achieved remission (DAS28 ESR <2.6) in the TCZ groups compared with corresponding TNFi groups (DMARD-IR, TCZ 44.0 % vs. TNFi 29.6 %; monotherapy, TCZ 37.2 % vs. TNFi 30.2 %; TNF-IR, TCZ 41.3 % vs. TNFi 19.2 %; *p* < 0.001 for all comparisons). More patients achieved moderate–good responses (EULAR criteria) in the TCZ treatment groups (79–85 %) compared with TNFi treatment groups (65–81 %). Patient-reported outcomes showed greater improvements in TCZ compared with TNFi groups. In patients with inadequate response to DMARDs and/or TNFi treated in routine clinical practice, TCZ in combination with DMARDs or as monotherapy resulted in significantly more patients achieving remission and more marked improvements in patient-reported outcomes compared with TNF inhibitors.

## Introduction

Rheumatoid arthritis (RA) is a common chronic inflammatory disease associated with progressive joint destruction, pain, fatigue and disability. Current treatments target the inflammatory response using disease-modifying anti-rheumatic drugs (DMARDs) and biological agents, in combination or as monotherapy. Five classes of biologics with differing modes of action are currently used in the treatment of RA: tumour necrosis factor inhibitors (TNFi) (adalimumab, certolizumab, etanercept, golimumab and infliximab), an interleukin (IL)-1 receptor antagonist (anakinra), a selective T-cell costimulatory modulator (abatacept), a chimeric anti-CD20 monoclonal antibody (rituximab) and a humanised anti–IL-6 receptor antibody (tocilizumab (TCZ)). Treatment guidelines recommend DMARDs (initially methotrexate [MTX]) as immediate first-line therapy in patients with RA, followed by combination therapy with conventional DMARDs, or with biological agents in combination with DMARDs, should patients fail to achieve remission or low disease activity on DMARDs alone [1, 2]. Monotherapy with biologics is not generally recommended for all agents in current guidelines, although in German guidelines, TCZ is recommended as monotherapy particularly in patients with intolerance to MTX or when the continuation of MTX therapy is considered inappropriate for other reasons [3]. The majority of available data on biologics in RA have been from studies of TNFis, and although shown to be more effective in combination with MTX than MTX alone, TNFi monotherapy was also shown to be less effective than combination therapy [4–8]. In more recent studies, TCZ has been shown to be effective both in combination with DMARDs and also as monotherapy in patients who had previously had an inadequate response to DMARDs (DMARD-IR) [9–13] or to TNF inhibitors (TNFi-IR) [14–16]. Most recently, the large Phase IV ADACTA trial demonstrated superior efficacy for monotherapy with TCZ as compared to monotherapy with the TNFi adalimumab in patients who were intolerant to, or unsuitable for, treatment with methotrexate [17]. However, such suitably powered randomised clinical head-to-head trials for biologics in RA are rare. Due to strict inclusion and exclusion criteria, a randomised trial might not reflect clinical practice; therefore, retrospective analyses of data from registries and chart-based analyses of patients treated in routine clinical practice may help guide decision-making for clinicians managing RA patients.

The current study therefore aimed to assess the efficacy of TCZ as compared with TNF inhibitors, alone or in combination with DMARDs, in patients with an inadequate response to prior DMARDs and/or TNFi managed in routine clinical practice.

## Materials and methods

This was a retrospective analysis of data from patients with RA managed in centres (office-based practices and clinics) throughout Germany. Centres that had treated at least three suitable patients in each specified TCZ and TNFi treatment group within the time period specified below were eligible to take part in the study. Patients who had inadequate response (IR) to previous treatment with either DMARDs (DMARD-IR) or with a TNFi (TNF-IR), or a combination of both, and who subsequently initiated treatment with either TCZ or a TNFi between 1 January 2010 and 31 December 2011 were eligible for inclusion. No other inclusion or exclusion criteria were specified. Collection of patient data was performed between January and May 2012. All data were fully anonymised; therefore, no patient consent was required for this analysis. Due to the non-interventional and anonymised nature of this analysis, no ethical approval of this study was required according to German drug law.

Data for eligible patients were collected via online case report forms (CRFs). For quality assurance, automatic online queries were generated in the CRF where data input appeared to be anomalous. Baseline demographics data, including sex, age, employment status, comorbidities, RA duration and treatment history at initiation of TCZ or the reference TNF inhibitor were collected. In addition, the following data were collected at baseline (defined as initiation of TCZ or reference TNFi) and week 12 of treatment where available: laboratory data including rheumatoid factor (RF) and anti-cyclic citrullinated peptide antibody (ACCP), haemoglobin, C-reactive protein (CRP) and erythrocyte sedimentation rate (ESR); and clinical assessments including swollen joint count (SJC) and tender joint count (TJC), patient- and physician-reported global health assessment by visual analogue scale (VAS), morning stiffness (minutes as assessed by patient) and corticosteroid requirement. Disease activity score 28 joint (DAS28), clinical disease activity score (CDAI) and simplified disease activity score (SDAI) were calculated automatically. The occurrence of adverse events (AEs) was recorded in a simplified manner as having been experienced or not experienced; no detailed description of the type or nature of AEs was collected. All collected data were analysed as observed; missing data were treated as treatment failures.

### Outcomes

The main outcome parameters were remission, defined as DAS28 (ESR) <2.6, and European League Against Rheumatism (EULAR) response, 3 months after initiation of treatment with TNFi or TCZ.

Patients were divided into six groups based on their treatment history and treatment initiated during the study period:

1. DMARD-IR patients initiating treatment with TCZ + DMARD (DMARD-IR TCZ)
2. DMARD-IR patients initiating treatment with a TNFi + DMARD (DMARD-IR TNFi)
3. DMARD-IR and/or TNFi-IR patients initiating treatment with TCZ monotherapy (TCZ mono)
4. DMARD-IR and/or TNFi-IR patients initiating treatment with TNFi monotherapy (TNFi mono)
5. TNFi-IR patients initiating treatment with TCZ + DMARD (TNFi-IR TCZ)
6. TNFi-IR patients initiating treatment with TNFi + DMARD (TNFi-IR TNFi)

### Statistics

Summary statistics (mean, median, standard deviation, 25th percentile, 75th percentile, minimum, maximum, number of values) or frequencies and proportions were assessed for all collected parameters. Independent two-sample *t* test with Levene’s test for equality of variances and *t* test for equality of means was used. Significance level was *p* < 0.05.

## Results

In total, 1603 patients from 70 centres were included in the study. Of these, 95 % were from community-based practices and 5 % from RA clinics. Baseline demographics are shown in Table 1. TNFis initiated during the observational period were as follows: adalimumab (39.5 %), etanercept (29.0 %), certolizumab pegol (14.7 %), golimumab (12.9 %) and infliximab (3.8 %).

**Table 1:** Baseline demographics

	DMARD-IR		DMARD-IR and/or TNF-IR		TNF-IR	
	TCZ + DMARD (*n* = 250)	TNFi + DMARD (*n* = 286)	TCZ mono (*n* = 288)	TNFi mono (*n* = 272)	TCZ + DMARD (*n* = 259)	TNFi + DMARD (*n* = 248)
Female/male, %	72/28	71/29	83/17	82/18	76/24	75/25
Age, years ± SD	57 ± 12	55 ± 12	58 ± 14	58 ± 13	56 ± 12	55 ± 12
RA duration, years ± SD	9 ± 8	9 ± 8	12 ± 9	11 ± 9	12.5 ± 10	11 ± 8
Employment status, %						
Full employment	80.6	85.2	63.9	76.9	58.3	72.6
Invalidity retirement due to RA	17.5	11.3	28.6	19.8	38.8	22.6
Previous therapy^a^						
Methotrexate^b^, %	76	74	38	40	74	72
Leflunomide^b^, %	29	32	25	30	17	23
Number DMARDs	2.2	2.2	2.7^**^	2.4^**^	2.5	2.4
Number Biologicals	0	0	1.3^***^	0.5^***^	1.6^***^	1.2^***^
Corticosteroid equivalent, mg/day	7.7	8.4	8.3	7.9	7.5	7.9
RF/ACPA positive, %	55/55	53/49	59/54	58/51	55/51	56/48
Number co-morbidities	1.88	1.84	2.02	1.86	1.80	1.94
Clinical parameters						
SJC	7.2 ± 4.9	6.9 ± 4.7	6.8 ± 4.8^*^	5.6 ± 4.2^*^	6.3 ± 4.2	5.9 ± 4.9
TJC	9.0 ± 5.6	8.7 ± 5.9	9.1 ± 6.2^*^	7.7 ± 5.3^*^	8.0 ± 5.4	7.7 ± 5.6
ESR, mm/h	36 ± 24^*^	31 ± 22^*^	36 ± 25	33 ± 23	37 ± 24^**^	33 ± 22^**^
CRP, mg/L	25 ± 36^***^	17 ± 24^***^	24 ± 33	16 ± 21	23 ± 30^**^	18 ± 22^**^
Morning stiffness (minutes)	69 ± 49	66 ± 50	68 ± 50^*^	60 ± 47^*^	74 ± 56^**^	61 ± 48^**^
VAS PGH	61 ± 21	55 ± 23	63 ± 20	55 ± 22	63 ± 19	59 ± 20
VAS PGA	58 ± 19	54 ± 21	61 ± 18^*^	53 ± 19^*^	60 ± 17	56 ± 18
DAS28 (ESR)	5.4 ± 1.3	5.2 ± 1.2	5.4 ± 1.3^***^	5.1 ± 1.2^***^	5.3 ± 1.2	5.1 ± 1.3
DAS28 (CRP)	5.0 ± 1.1^*^	4.8 ± 1.1^*^	5.0 ± 1.1^***^	4.6 ± 1.1^***^	4.9 ± 1.0	4.7 ± 1.2
CDAI	30 ± 13	28 ± 12	31 ± 12^***^	25 ± 11^***^	29 ± 11^*^	27 ± 13^*^
SDAI	28 ± 11	26 ± 11	28 ± 11^***^	24 ± 10^***^	27 ± 10	25 ± 12

*ACPA* anti-cyclic citrullinated peptide antibody, *CDAI* clinical disease activity score, *CRP* C-reactive protein, *DAS28* disease activity score 28 joint, *ESR* erythrocyte sedimentation rate, *PGA* physician global assessment, *PGH* patient global health, *RF* rheumatoid factor, *SDAI* simplified disease activity score, *SJC* swollen joint count, *TJC* tender joint count, *VAS* visual analogue scale

*p < 0.05; **p < 0.01; ***p < 0.001

^a^Multiple answers possible

^b^In the 6 weeks prior to baseline

The TCZ and TNFi groups were comparable with regard to age and gender distribution, and disease duration. However, patients initiating treatment with TCZ generally had more severe disease than those in the corresponding TNFi group (e.g. higher SJC, TJC, morning stiffness, DAS28, and patient and physician VAS scores). Significantly more patients in the DMARD-IR TCZ group and the TCZ mono group had DAS28 (ESR) >5.1 at baseline compared with the corresponding TNFi groups (65 vs. 55 %, and 65 vs. 54 %, respectively; *p* < 0.001). In addition, the proportion of patients with invalidity retirement due to RA was higher in the TCZ groups. TCZ patients in both the monotherapy and TNFi-IR group had been more extensively pre-treated compared with patients initiating a TNFi and had received significantly more prior treatment with biologicals (Table 1).

The number and nature of comorbidities were generally similar between TCZ and TNFi groups, with the exception of the monotherapy groups, where patients initiating TCZ monotherapy had a higher proportion of comorbidities compared with those initiating TNFi monotherapy (Table 2).

**Table 2:** Comorbidities (% of patients)

	DMARD-IR		Mono		TNF-IR	
	TCZ	TNFi	TCZ	TNFi	TCZ	TNFi
Osteoporosis	33.2	34.3	36.5	36.0	31.7	33.1
Hypertension	28.4	33.2	38.9	31.2	30.1	38.7
Obesity	22.0	19.9	19.1	17.6	25.5	23.4
Anaemia	18.8	17.6	20.8	14.6	15.6	12.6
Lipid metabolism disorder	12.8	9.4	10.4	7.7	8.9	6.9
Diabetes mellitus	11.2	8.0	13.5	8.8	11.2	11.7
COPD/asthma	8.0	5.6	5.9	8.1	6.5	8.4
Depression (diagnosed)	3.6	4.9	5.2	3.3	3.5	5.2
Depression (suspected)	6.0	3.5	3.8	2.9	3.5	2.0
Coronary heart disease	3.2	4.6	2.8	3.3	5.0	2.8
Hypothyroidism	4.8	2.4	4.2	5.1	4.2	4.0
Hyperthyroidism	3.2	2.4	1.7	1.5	2.7	4.4

The reasons for the use of monotherapy given most commonly by clinicians were (multiple reasons could be given) the following: efficacy of monotherapy (TCZ 56 %, TNFi 44 %), intolerance to prior therapy (TCZ 48 %, TNFi 52 %), compliance issues (TCZ 52 %, TNFi 48 %), contraindications (hepatic, TCZ 40 %, TNFi 60 %; renal, TCZ 11 %, TNFi 89 %), de-escalation of therapy e.g. scaling down from combination to monotherapy (TCZ 42 %, TNFi 58 %).

### Efficacy

**Fig. 1:**
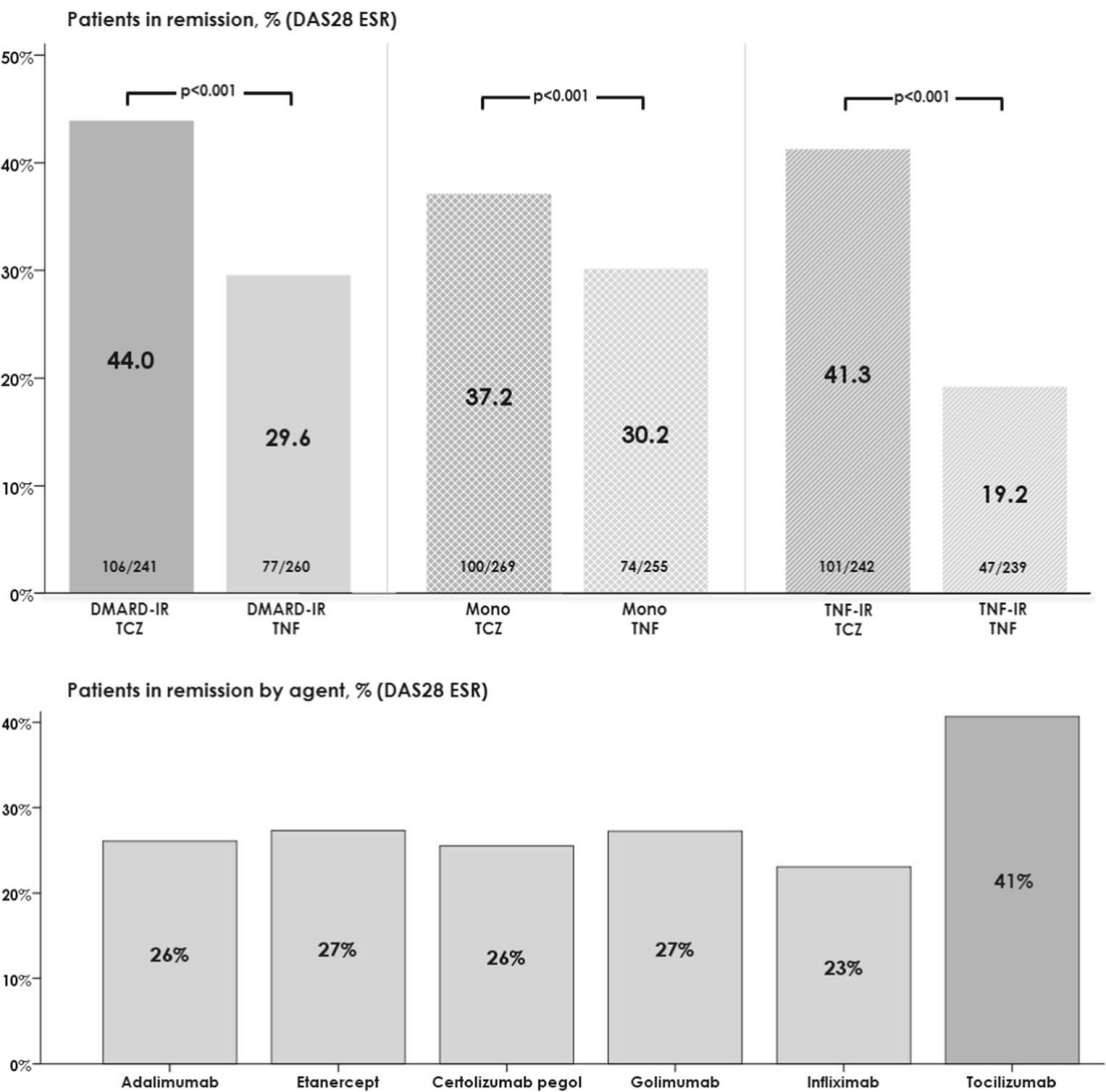
DAS28 remission at week 12. DAS28 (ESR) < 2.6. *DAS28* disease activity score 28 joint, *DMARD* disease-modifying anti-rheumatic drugs, *ESR* erythrocyte sedimentation rate, *IR* insufficient response, *TCZ* tocilizumab, *TNFi* tumour necrosis factor inhibitor

The proportion of patients achieving remission (DAS28 ESR <2.6) was significantly higher in the TCZ groups compared with corresponding TNFi groups (Fig. 1a). Overall, the proportion of patients who achieved remission at week 12 was similar between the different TNFis and was higher in patients treated with TCZ (Fig. 1b).

**Fig. 2:**
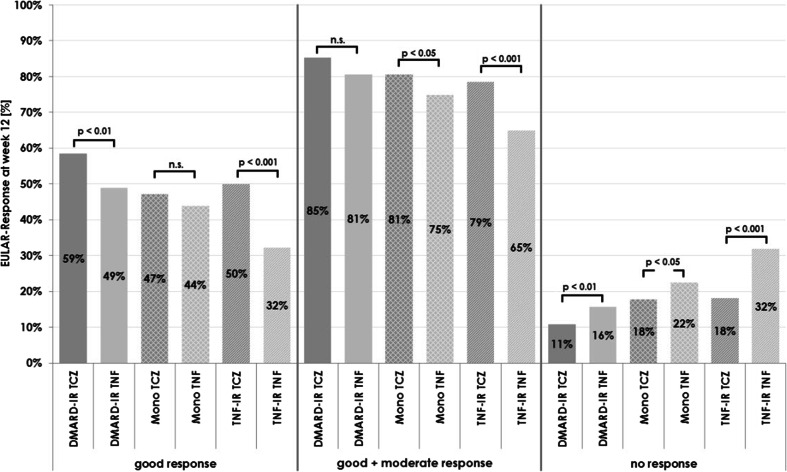
EULAR-Response at week 12 by EULAR criteria. *n.s.* not significant, *DMARD* disease-modifying anti-rheumatic drugs, *EULAR* European League Against Rheumatism, *IR* insufficient response, *TCZ* tocilizumab, *TNF-i* tumour necrosis factor inhibitor

The proportion of patients achieving moderate-to-good or good responses according to EULAR criteria was higher in the TCZ treatment groups compared with the corresponding TNFi treatment groups (Fig. 2). In agreement with this, the proportion of patients who failed to respond to therapy was higher in the TNFi treatment groups compared with the corresponding TCZ treatment groups (Fig. 2). Non-response led to treatment discontinuation in 4.4 % of patients treated with TCZ and 12.2 % of patients treated with TNFi. It should be noted that achieving a ‘moderate response’ by EULAR criteria was sufficient for some patients to enter remission.

The proportion of patients achieving low disease activity (DAS28 ESR ≤3.2) at week 12 was significantly greater in the TCZ treatment groups compared with the corresponding TNFi groups (DMARD-IR TCZ 64 %; DMARD TNFi 50 %; mono TCZ 51 %; mono TNFi 45 %; TNF-IR TCZ 60 %; TNF-IR TNFi 36 %; *p* ≤ 0.01 for all comparisons).

**Fig. 3:**
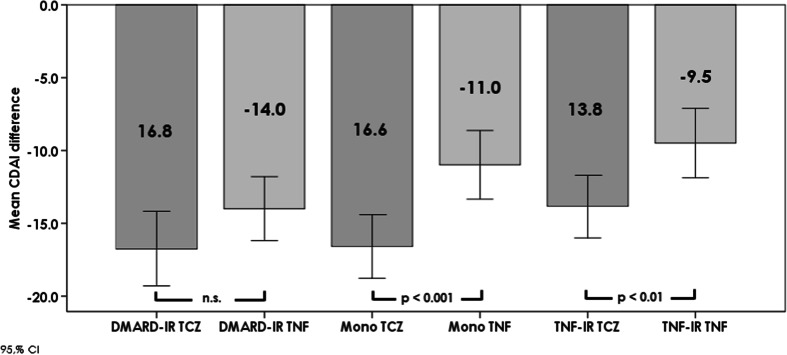
Mean change in CDAI (95 % CI) at week 12 vs. baseline by treatment group. *n.s.* not significant, *CDAI* clinical disease activity score, *DMARD* disease-modifying anti-rheumatic drugs, *IR* insufficient response, *TCZ* tocilizumab, *TNFi* tumour necrosis factor inhibitor

Patients treated with TCZ also showed greater improvements in CDAI score at week 12 of treatment vs. baseline compared with those treated with TNFi, and this reached statistical significance in the monotherapy and TNFi-IR groups (Fig. 3). There was no significant difference between TCZ and TNFi arms with regard to change in SDAI with the exception of the monotherapy arms where patients treated with TCZ showed significantly greater improvements (data not shown).

The majority of patients included in the study were able to reduce their steroid use over the 12-week treatment period (80 % in the TCZ groups and 70 % in the TNFi groups; *p* = non-significant). Overall, the mean reduction in steroid reduction was not significantly different between groups, with the exception of the TCZ and TNFi monotherapy groups, where mean dose reduction was higher in the TCZ group (mean change [dose equivalent, mg/day]: DMARD-IR TCZ −2.5; DMARD TNFi −2.4; mono TCZ −2.5; mono TNFi −0.6; TNF-IR TCZ −1.6; TNF-IR TNFi −2.0).

**Fig. 4:**
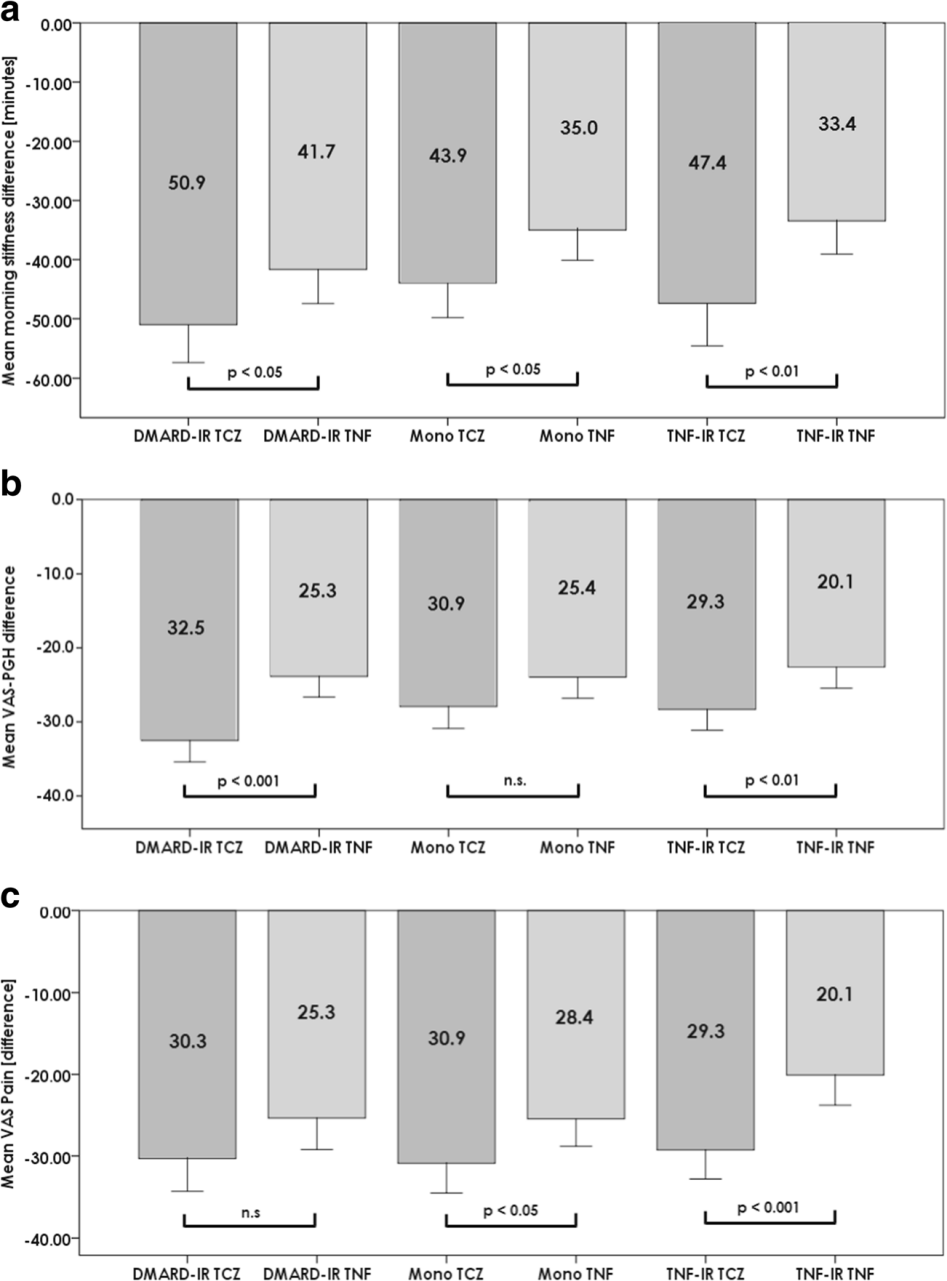
Patient-reported outcomes at baseline and week 12 by treatment group: **a** Morning stiffness, **b** VAS patient global health (100 units) and **c** VAS Pain (100 units). *n.s.* not significant, *DMARD* disease-modifying anti-rheumatic drugs, *IR* insufficient response, *PGH* patient global health, *TCZ* tocilizumab, *TNFi* tumour necrosis factor inhibitor, *VAS* visual analogue scale

Patient-reported outcomes improved in all treatment groups (Fig. 4a–c). Overall, there were greater improvements in morning stiffness, patient global health and pain with TCZ compared with TNFi in all groups indicating the better reduction of disease activity by TCZ.

### Safety and tolerability

Overall, 4.8 % of patients in the TCZ groups and 3.2 % of patients in the TNFi groups experienced treatment-associated adverse events (AEs). No serious AEs were reported. Rates of treatment discontinuation due to AEs were low in all groups (overall rate 3 % in the TCZ groups vs. 1 % in the TNFi groups). Although further details of AEs as reported to Roche as part of post-marketing safety surveillance were available, no such data were available for TNFi; therefore, no further comparisons are possible.

## Discussion

In this large cohort of patients with inadequate response to DMARDs and/or TNFi managed in routine clinical practice, patients treated with TCZ alone or in combination with DMARDs had significantly higher rates of remission (DAS28 < 2.6) compared with patients treated with similar regimens using TNFi. Treatment with TCZ was also associated with higher rates of good or moderate EULAR response and lower rate of non-response compared with TNFi and significantly greater improvements in CDAI. Improvements in patient-reported outcomes such as morning stiffness and pain also tended to be greater in patients treated with TCZ compared with those treated with TNFi.

The greater efficacy of TCZ compared with TNFi was apparent despite the fact that patients in the TCZ groups generally had more severe disease and had been more intensively pre-treated compared with those in the corresponding TNFi groups. Overall, our data suggests that patients in the TCZ treatment groups had a history of greater disease impairment, with fewer patients in full-time employment and more patients having entered invalidity retirement due to RA. This may be a reflection of EULAR treatment guidelines in place at the time of the study which recommend that TNFi should be the first biologic DMARD used (in combination with MTX) for patients who fail to respond to conventional DMARDs; current guidelines do not specify [2]. Loss of work productivity occurs early in the course of the disease and contributes greatly to the overall costs of RA [9, 17]. In a Swedish study, work disability made up the largest cost of RA, exceeding the costs associated with treatment [18]. Achievement of disease remission has been shown to be associated with lower number of days lost from work due to sick leave and permanent work disability [19]. Available data suggest a decrease in work disability in patients with RA over recent years which may be associated with more active treatment strategies and more specifically the introduction of biologics [20–22].

Although achieving disease control and thereby prevention of structural damage is the primary goal when treating RA, other aspects of the disease may be more important to the patient. Morning stiffness and pain have been shown to be closely related to functional disability [23] and to have adverse effects on quality of life, ability to work and the need for early retirement due to RA [24, 25]. Improvements in these parameters are important as these debilitating symptoms can occur even in patients with apparently well-controlled RA [26]. In our cohort, there was an improvement in patient-reported outcomes including morning stiffness, global health and pain after 12 weeks of treatment in all patient groups. Change from baseline was numerically and generally significantly greater in TCZ-treated patients. These patients were generally more symptomatic at baseline compared with the corresponding TNFi treatment group, which potentially might have contributed to the greater overall change seen. Patients with RA have been shown to have raised serum concentrations of pro-inflammatory cytokines compared with normal controls which correlate with disease activity. However, while levels of TNF do not change significantly during the overnight period, patients with active RA have been found to have elevated levels of IL-6 during the early morning, which are linked to morning stiffness and pain [27]. The potential ability of TCZ to block this circadian increase in IL-6 may contribute to its better efficacy in reducing these symptoms compared with TNFi seen in our study. However, as morning stiffness is related to a range of features other than IL-6 levels, such as disease activity level and general health [28], other factors may also contribute. It would be of interest to investigate other patient-reported outcomes. However, the collection of data on measures such as the Health Assessment Questionnaire (HAQ) and the Funktionsfragebogen Hannover (FFbH) is not routinely collected in Germany and was performed at the discretion of clinician in the current study. Although a limited number of these data were available, they were too small to allow statistical comparison.

As this study was not a controlled head-to-head comparison of TCZ and TNFis, comparative data should be treated with caution. Although several studies have compared biologics with different modes of action in patients with RA, to date, only two trials in DMARD-IR patients have included an adequately powered head-to-head comparison. The AMPLE trial compared abatacept, a selective T-cell costimulatory modulator and the TNFi adalimumab, both in combination with a stable dose of methotrexate, and found no difference in efficacy between the two agents based on clinical, functional and radiographic outcomes [29]. In contrast, in the ADACTA trial which compared TCZ monotherapy with adalimumab monotherapy in MTX-ineligible patients with severe RA, TCZ was found to be more effective than the comparator, with significantly greater improvements in DAS28 (−3.3 vs. −1.8, respectively; *p* < 0.0001), and significantly more patients achieving remission (DAS < 2.6: 39.9 vs. 10.5 %, respectively; *p* < 0.0001), as well as EULAR good or moderate (77.9 vs. 54.9 %; *p* < 0.0001) and good responses (51.5 vs. 19.8 %; *p* < 0.0001) [17]. The rates of remission and response in the current observational study were somewhat higher than those obtained in ADACTA, possibly reflecting the fact that patients in the latter trial had more severe RA overall than those in our trial (DAS28 6.7 in ADACTA vs. around 5.3 in the current study) and the patients treated with monotherapy in CONSENS were DMARD and/or TNF-IR. Although the use of biologics as monotherapy is not generally recommended by guidelines, studies suggest that it still is widely used in routine clinical practice [23, 30]. A recent ‘real-world’ study found that TCZ monotherapy was more effective in terms of reducing DAS28 and was better tolerated compared with TNFi in 254 RA patients treated in 30 centres in Germany [30]. Our study therefore supports and adds to the available data on the use and efficacy of TCZ and TNFi as monotherapy in routine practice and also provides additional data on real-world efficacy of combination therapy in both DMARD-IR and TNFi-IR patients.

One of the main advantages of the current study is the enrolment of a large number of patients from an extensive range of clinical practices. Despite obvious limitations compared with prospective studies, retrospective studies provide an important source of information on the efficacy of treatment under real-life conditions. The simple inclusion criteria used in the current study ensured that a wide range of RA patients were included, more closely reflecting typical patients encountered in daily practice as compared with those in clinical trials, which have highly restrictive inclusion and exclusion criteria.

This study has the usual limitations related to its retrospective, uncontrolled nature. Although participating centres were encouraged to include all eligible patients on their lists, selection bias cannot be excluded. Choice of therapy was at the discretion of the clinician; therefore, selection bias for treatment choice also cannot be ruled out. Only data actually recorded were available for inclusion. Not all patients had results for all parameters at each time point, and this was particularly notable for some parameters which are not routinely performed in day-to-day practice in Germany. Missing data may therefore potentially have affected overall results. Detailed data on adverse events (AEs) were not recorded; although the numbers of AEs reported were similar between TCZ- and TNFi-treated patients, no information on the nature or severity of AEs was available. Finally, we selected the study period to reflect EULAR guidelines which recommend the main efficacy assessment 12 weeks after therapy initiation or adjustment. Data are therefore relatively short-term, and longer-term studies are required to confirm if the improvements seen at 12 weeks are sustained over long-term treatment.

In summary, in DMARD-IR and TNFi-IR patients treated in routine clinical practice, TCZ in combination with DMARDs or as monotherapy resulted in significantly more patients achieving remission compared with TNF inhibitors, although TCZ-treated patients had more severe disease of longer duration at baseline. In addition, significantly greater improvements were seen in patient-reported outcomes such as morning stiffness and pain in TCZ-treated patients. Fewer patients treated with TCZ discontinued treatment due to non-response.
